# Plasma optics improving plasma accelerators

**DOI:** 10.1038/s41377-022-00925-2

**Published:** 2022-07-29

**Authors:** Andreas Döpp

**Affiliations:** Ludwig-Maximilians-Universität München, Garching, Germany

**Keywords:** Plasma-based accelerators, Laser-produced plasmas

## Abstract

Plasma accelerators driven by high-power lasers can provide high-energy electron beams on a dramatically smaller scale than conventional radio-frequency accelerators. However, the performance of these accelerators is fundamentally limited by the diffraction of the laser. Laser-generated plasma waveguides can mitigate this problem and, combined with a controlled injection method for electrons, highlight the potential of novel laser-plasma optics.

Modern laser systems with peak powers at the terawatt or even petawatt level have unlocked access to entirely new regimes of optics, which have been coined *relativistic* optics^1^ or *plasma* optics^2^. When focused onto a micrometer spot size, the field gradients within the laser can reach many teravolts per meter, which is enough to accelerate electrons to near lightspeed within a single cycle. Not only are these particles immediately ionized and form a plasma, but their increased momentum also modifies the refractive index of the medium. In a plasma at atmospheric density the laser is locally slowed down, curving its wavefront to focus. This relativistic self-focusing counteracts natural diffraction and allows the laser pulses to maintain their extremely high intensity over several Rayleigh lengths^1^.

Meanwhile, the interplay between excited electrons and quasi-stationary ions leads to the formation of plasma oscillations in the wake of the laser pulses. These wakefields possess very high fields of their own. But in contrast to the transversely oriented laser fields, the plasma wakefields contain a strong longitudinal component (~100 GV/m). This is many orders of magnitude more than accelerators run at radio-frequency achieve and a laser wakefield accelerator (LWFA) can accelerate electrons to GeV energies within a centimeter^3,4^.

The energy gain in LWFA is limited by the “three D’s”, namely the available laser energy (depletion), the phase slip between electrons and the wakefield-exciting laser (dephasing) and the intensity of the laser (diffraction). To reduce dephasing one has to lower the plasma density, but this in turn weakens the self-focusing effect significantly. To overcome this deadlock one needs to guide the laser in some alternative manner. This was first successfully demonstrated using discharge-generated plasma channels^5^, but these devices have only found limited use due to their complexity. More recently, all-optical techniques have gained traction, when several groups demonstrated efficient guiding in plasma channels generated by Bessel beams^6–8^. Conveniently, these line-focused guiding beams can often be derived from parts of the high-power laser that drives the plasma accelerator.

Now, writing in this issue of *Light: Science & Applications*, a team of scientists from Laboratoire d’Optique Appliquée in France has successfully combined an all-optical plasma waveguide with a state-of-the-art laser wakefield accelerator^9^. The guiding channel is generated by a 1.5 mJ, 30 fs laser pulse, which is intense enough to create a plasma via field ionization. The plasma then expands radially, forming a waveguide. After 2 nanoseconds, the thousand times more powerful 50 TW drive beam is sent into the plasma and drives a plasma wakefield, as sketched in Fig. 1.

**Fig. 1:**
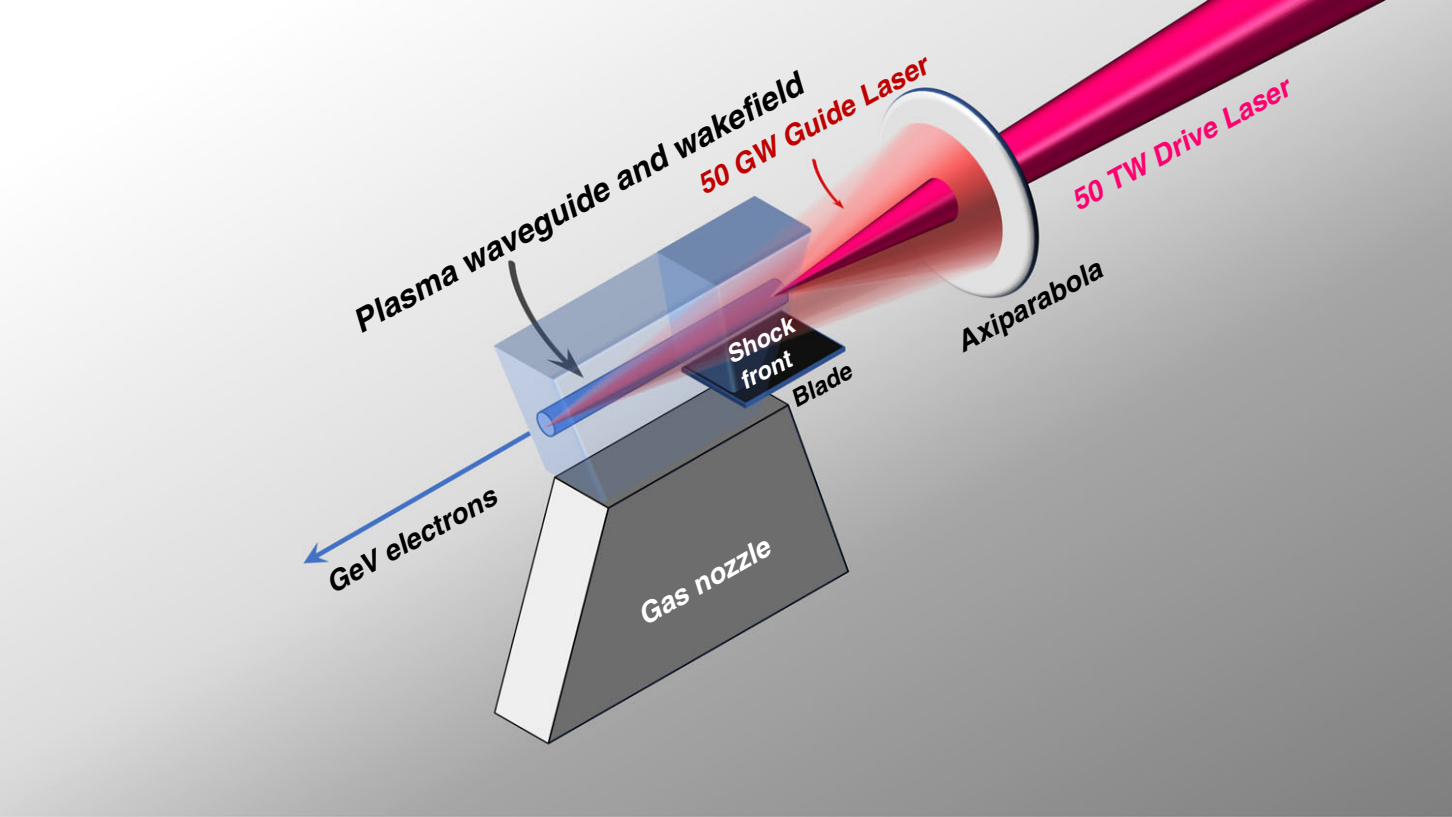
Schematic view of the experimental setup for plasma waveguide and laser-driven wakefield acceleration

The authors find that the drive beam can be efficiently guided over 15 mm, which is about 20 times the Rayleigh length of the focused laser in vacuum. Maintaining high intensity over this distance allowed the laser wakefield to accelerate electrons to GeV-level energy, yet with significant energy spread. Similar results were recently reported by a group of researchers from the United Kingdom and the United States, who used an even more powerful 300 TW laser and reached beam energies of up to 4 GeV^10^.

In order to achieve the small energy spread required in many applications, the team under the direction of Dr. Cedric Thaury not only shaped the radial plasma profile but added tailoring of the density profile along the longitudinal direction. This was achieved by locally obstructing the flow from their gas jet target. The technique, commonly referred to as “shock-front injection”^11^, facilitates electron injection in a very localized region of the plasma and is known to produce beams with much smaller energy spread. Using this combined radial and longitudinal plasma tailoring, the authors report beams with 2–4 percent energy spread at energies around 1 GeV. While the energy varies from shot to shot, this is in large part correlated with electron beam charge, pointing at beamloading as a main cause for the observed fluctuations^12^.

The results are an excellent example for the potential of all-optical plasma shaping to generate optical elements that can withstand the highest field intensities. Beyond plasma mirrors^13^, holograms^14^ and the waveguides discussed in this article, lasers can also be used for longitudinal plasma shaping^15^, which would allow for all-optical guiding, injection, and acceleration.
